# Topological View of Flows Inside the BOLD Spontaneous Activity of the Human Brain

**DOI:** 10.3389/fncom.2020.00034

**Published:** 2020-04-22

**Authors:** Arjuna P. H. Don, James F. Peters, Sheela Ramanna, Arturo Tozzi

**Affiliations:** ^1^Computational Intelligence Laboratory, University of Manitoba, Winnipeg, MB, Canada; ^2^Applied Computer Science, University of Winnipeg, Winnipeg, MB, Canada; ^3^Department of Physics, University of North Texas, Denton, TX, United States

**Keywords:** Betti Numbers, brain activity, fMRI video, persistence bar code, topological data analysis

## Abstract

Spatio-temporal brain activities with variable delay detectable in resting-state functional magnetic resonance imaging (rs-fMRI) give rise to highly reproducible structures, termed cortical lag threads, that propagate from one brain region to another. Using a computational topology of data approach, we found that persistent, recurring blood oxygen level dependent (BOLD) signals in triangulated rs-fMRI videoframes display previously undetected topological findings, i.e., vortex structures that cover brain activated regions. Measure of persistence of vortex shapes in BOLD signal propagation is carried out in terms of Betti numbers that rise and fall over time during spontaneous activity of the brain. Importantly, a topology of data given in terms of geometric shapes of BOLD signal propagation offers a practical approach in coping with and sidestepping massive noise in neurodata, such as unwanted dark (low intensity) regions in the neighborhood of non-zero BOLD signals. Our findings have been codified and visualized in plots able to track the non-trivial BOLD signals that appear intermittently in a sequence of rs-fMRI videoframes. The end result of this tracking of changing lag structures is a so-called persistent barcode, which is a pictograph that offers a convenient visual means of exhibiting, comparing, and classifying brain activation patterns.

## 1. Introduction

**Figure 1:**
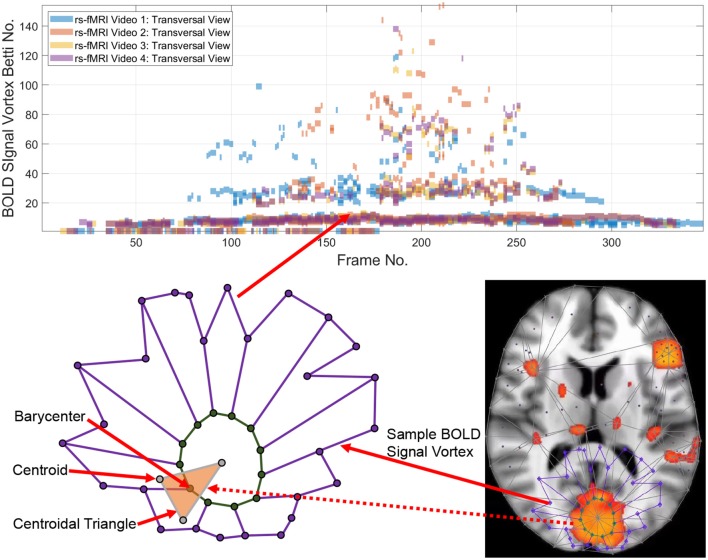
Betti numbers for a rs-fMRI BOLD signal vortex on a Transversal view for four videos.

Point clouds are a natural outcome of a topology of data approach in tracking intermittent as well as persistent BOLD signals in different sections of the brain. A point cloud is a collection of sampled pinpointed places in a subregion (Ghrist, 2014). A topology of data circumvents noise in data and focuses on those data that persist over time (Edelsbrunner and Harer, 2010). Computational topology of data provides a practical method in isolating, measuring, and classifying persistent lag structures BOLD signals in each rs-fMRI video frame that are mapped to point clouds in a finite, bounded region in an *n*-dimensional Euclidean metric space. It has been observed that brain activity in one region of the brain propagates to others with variable temporal delay (Mitra et al., 2015; Matsui et al., 2016; Park et al., 2019), giving rise to brain activity lag (delay) structures. Lag threads are temporal sequences of propagated brain activities (Mitra et al., 2015). Lag structures in fMRI video frames are rich source of point clouds. Triangulated brain point clouds are a source of brain activation area shapes that appear intermittently in different cortical regions during a rs-fMRI video such as the four videos provided by Mitra et al. (2015). Selected barycenters of triangles in a triangulated cortical point cloud are connected to form vortexes covering each brain activation region with its own distinctive shape. Each vortex in a triangulated rs-fMRI video frame is a collection of connected cycles (see, e.g., the lower half of Figure 1) that make it possible to approximate, measure, track and compare brain activation region shapes. A vortical view of brain activity first appeared in Freeman (2009).

Each vertex in a triangulated brain activation region point cloud is represented by a feature vector containing useful information such as activation region area and representative Betti number (Giusti et al., 2015). Traditionally, the more intuitive geometric forms of Betti numbers are counts of cells (vertexes, edges, filled triangles), cycle counts or surface hole counts (Zomorodian, 2001). A particularly useful intuitive form of geometric Betti number is a count of the number of connected vortex cycles covering an activation subregion (Peters, 2020). Less frequently used algebraic Betti numbers (counts of the number of generators in a free Abelian group; Munkres, 2000) also provide insight concerning the inner workings of cycles in triangulated brain activation regions. We first consider the persistence of geometric numbers (vortex cycle counts) over sequences of sequences of triangulated rs-FMRI video frames. Later, in Appendix B, the persistence of algebraic Betti numbers is also considered to obtain an alternative view of the changing character of connected cycles in triangulated rs-FMRI lag threads in three regions of the brain.

Tracking the appearances of the Betti number of a brain activation vortex containing a particular number of connected cycles leads to the construction of a persistence barcode (see the top half of Figure 1) in which a Betti number appears in a video frame, disappears afterward and possibly reappears one or more times in later video frames. 2D (planar) as well as 3D (volumetric) persistent barcodes provide an easy-to-read means of tracking intermittent BOLD signals in a sequence of rs-fMRI video frames.

The origin of topological data analysis and persistent homology can be traced back to Edelsbrunner et al. (2000, 2001). A common approach is to build a continuous shape (graphs) on top of data to detect complex topological and underlying geometric structures (Carlsson, 2009; Chazal and Michel, 2017). This shape is called a simplicial complex or a nested family of simplicial complexes and the process of shape construction is commonly referred to a filtration (Zomorodian, 2001). One of the fundamental tools in computational topology is persistent homology (Zomorodian and Carlsson, 2005), which is a powerful tool to compute, study, and efficiently encode multiscale topological features of nested families of simplicial complexes and topological spaces (Edelsbrunner and Harer, 2008).

Earlier studies of brain networks primarily focused on a graph-theoretic approach where the brain regions and their connections are encoded as a graph (i.e., a network of nodes and edges) and cycles (representing complex behaviors). Such networks were modeled and analyzed with methods such as Q-modularity (Meunier et al., 2009) or with network measures such as betweeness centrality (Bullmore and Sports, 2009). Brain networks with weighted edges where problems of selecting thresholds for edge weights and dealing with sparse edges can be found in Achard and Bullmore (2007) and van Wijk et al. (2010). This has led to application of persistent homology to the problem of determining multiple thresholds derived from more than one network. A brain network can be considered as the 1-skeleton of a simplicial complex, where the 0-dimensional hole is the connected component, and the 1-dimensional hole is a cycle (Chung et al., 2019). The number of k-dimensional holes of a simplicial complex is its k-th Betti number. Persistent homology-based multiscale hierarchical modeling was proposed in Lee et al. (2011b), Petri et al. (2014), Giusti et al. (2015), Sizemore et al. (2018), and Chung et al. (2019) to name a few. Here, graph filtration is used to build these networks in a hierarchically manner. Filtration is the process of connecting edges to form a graph.

Betti numbers computed during this filtration process have been used for further statistical analysis such as Pearson correlation to MRI image data (Chung et al., 2015) and various metrics for similarity and distances assessments (Lee et al., 2011a; Chung et al., 2019). 0-dimensional holes (β_0_ or zeroth Betti numbers) have been computed during the graph filtration process and a persistent barcode has been constructed for subsequent statistical analysis (Cassidy et al., 2015; Chung et al., 2017). However, the *cycle* concept is extremely important to the study of behavior diffusion and integration of the brain network. In persistent homology, cycles are measured using the first or one-dimensional Betti Number (β_1_). In Chung et al. (2019), the one dimensional Betti numbers are used to measure cycle and the significance of the number of cycles is evaluated using the Kolmogorov–Smirnov (KS) distance.

For geometric representations of rs-fMRI lag threads, an incisive form of statistical analysis of the geometry is given in terms of edge density and Eigen values (Giusti et al., 2015). The results of this form of geometry-based statistical analysis are given in Appendix A.

In contrast to earlier approaches, we use computational geometry to detect lag thread shapes in fMRI video frames using a *geometric* Betti number that counts the total number of connected cycles forming a vortex (nested, usually non-concentric, connected cycles) derived from the triangulation of brain activation regions. We build on our previous work in Peters et al. (2017), Don and Peters (2019), and Peters (2020), to evaluate real BOLD resting state rs-fMRI videos from Mitra et al. (2015).

Here, the video frames are processed directly to obtain Betti numbers by triangulating the transversal, sagittal, and coronal sections of the fRMI video frames and constructing vortexes through a process of filtration. Rather than constructing graphs of brain networks and analysing cycles in the network, we analyze cycles by processing the video frames. The vortexes correspond to the changing activation areas in the video frames. The number of vortexes in a frame represents the most relevant areas of change. Higher Betti number values imply that the change is closer to the center of the section of the brain. To the best of our knowledge, this method of detecting cycles in persistent homology is novel and a preliminary version of this paper appeared in Don A. P. et al. (2019).

## 2. Materials and Methods

### 2.1. Theory

The basic approach is to introduce a geometric representation of brain activation regions in terms of intersecting cycles that are sequences of path-connected vertexes on the barycenters of triangles forming vortices (Don and Peters, 2019; Peters, 2020). Each vortex is a collection of connected cycles called a vortex nerve. Of particular interest are those nerves that have a maximal collection of triangles of a common vertex in the triangulation of a finite, bounded planar region. In our case, the planar region is a rs-fMRI video frame. A vortex nerve results from the triangulation of the sections of each rs-fMRI brain video frame. A centroid (also called a seed point), is used as a vertex in the triangulation of a video frame. A barycenter on a such a triangle is in a high light intensity video frame region between the dark regions, which we refer to as *holes*.

Definition 1 (Hole). A ***hole*** is a collection of contiguous low intensity voxels. The ***centroid of a hole*** is the center of mass of the hole. In an rs-fMRI video frame, the holes are in background regions containing dark (low intensity) voxels and the foreground regions are filled with mainly orange voxels. Sample centroids of brain activation region holes are given in Figure 1.

Using Delaunay triangulation (Delaunay, 1934; Yung et al., 2016), each pair of closest neighboring centroids of holes become the vertexes of edges of triangles covering brain activation regions in rs-fMRI videos. Each line segment drawn between closest pairs of centroids becomes the edge of a triangle in the Delaunay triangulation of an rs-fMRI video frame. The concept of a hole is crucial to this work, since edges drawn between barycenters in the interior of adjacent centroidal triangles reveal paths of high intensity voxels between brain activation region holes.

Definition 2 (Barycenter of centroidal triangle). Recall that the median line of a triangle is a line drawn from a vertex to the midpoint of the opposite side of the triangle. The ***barycenter of a centroidal triangle*** is the point of intersection of the median lines of the triangle. A sample barycenter is given in Figure 1.

The barycenters of centroidal triangles covering brain activation regions are always between holes. Barycenters are stepping stones in the construction vortex cycles.

Definition 3 (Barycentric cycle). A ***barycentric cycle*** is a sequence of edges drawn between neighboring barycenters of adjacent centroidal triangles.

As a result, connected barycenters model paths for high intensity voxels recorded in a brain activity video frame. Barycentric cycles are the basic building blocks in the construction of local vortexes covering triangulated brain activation regions.

Definition 4 (Local Vortex (briefly, Vortex)). A ***local vortex*** is a collection of nesting, usually non-concentric barycentric cycles. The simplest vortex contains a single barycentric cycle (see, for example, the vortex in Figure 3).

### 2.2. Betti Numbers on a Triangulated Brain Activation Region

Betti numbers provide a computational topology perspective on the structure of brain activation subregions. In our case, the ***Betti number*** β_1_ tells us either the number of connected vortex cycles in a vortex on a triangulated brain activation region (***geometric view***). Later, in Appendix B, we also introduce and apply the Betti number β_α_, which is a count of the number of generators in a free Abelian group representation of an rs-fMRI video frame vortex (***algebraic view***). The focus here is on the persistent geometric Betti numbers across sequences of triangulated video frames. Each such Betti number is mapped to an entry in a persistent barcode (see top half of Figure 1). This topology of data pictographs is useful in representing the persistence of the brain activation region shapes found in rs-fMRI brain video sections. From an intuitive perspective, there are three types of geometric Betti numbers, namely, β_0_, β_1_, β_2_, introduced in Zomorodian (2001).

Definition 5 (β_0_). The ***Betti number β*_0_** is a count of the total number of elementary cell complexes, which are vertexes, edges and filled triangles attached to each other in a triangulated region.

Definition 6 (β_1_). The ***Betti number β*_1_** is a count of the number cycles.

Each β_1_ in a persistent barcode (Ghrist, 2008) represents the number of connected barycentric cycles covering an activation area of the brain.

Definition 7 (β_2_). The ***Betti number β*_2_** is a count of the number of holes. In our case, **β_2_** is a count of the number of contiguous low intensity voxels in brain activation regions in an rs-fMRI video.

Definition 8 (*C*_0_ vortex cycle). ***cycle C*_0_** is the ***innermost cycle in a vortex***. In our case, the sequence of edges connected between barycenters of adjacent triangles with a common vertex, collectively called an Alexandroff nerve (Alexandroff, 1965) forms the *cycle* *C*_0_. Notice that cycle *C*_0_ will always be in the interior of a brain activation region containing high intensity voxels.

Definition 9 (*C*_1_). In a vortex with 2 nested cycles, ***cycle C*_1_** is the vortex cycle that has only *C*_0_ in its interior. In effect, cycle *C*_1_ is a collection of path-connected vertices on a sequence of edges surrounding *C*_0_. Cycle *C*_1_ usually overlaps the boundary of a high intensity brain activation region in an rs-fMRI video frame.

Definition 10 (Bridge Segment). A ***bridge segment*** is an edge attached between vertices on a pair of neighboring cycles. Let *cycA, cycB* be a pair of neighboring cycles (i.e., there are no cycles in between *cycA* and *cycB*) and let *p* be a vertex on *cycA* and *q*, a vertex on *cycB*. The edge $\bar{pq}$ is a bridge segment. Edge $\bar{cj}$ between vertices *c* and *j* is a bridge segment and there is no bridge segment between vertices *c* and *i* in Figure 5.

In the main body of this paper, we give barcoded video results for the more intuitive geometric Betti number counts of vortex cycles on triangulated brain activation regions. Later, in Appendix B, we give examples of both geometric and algebraic forms of Betti numbers. A repetition of the same β_1_ across a sequence of consecutive frames tells us that a similar vortex shape recurs in these frames. The geometric Betti number of a vortex containing two cycles with *k* bridge segments attached between the pair of vortex cycles equals k + 2 (Don and Peters, 2019).

For example, the Betti number β_0_ of the vortex in the lower half of Figure 1 equals 12 and β_1_ = 1, since there are 12 cycle edges and there is only one cycle in the vortex shown.

### 2.3. rs-fMRI Lag Threads Having Descriptive Proximity

A pair of objects are descriptively proximal (near each other), provided the objects have the same description (Di Concilio et al., 2018). A ***feature vector*** provides a description of an object. In this work, feature vector (Betti number, area) describes a subregion of a rs-fMRI brain region. Rather than a purely theoretical, abstract approach to descriptive proximity spaces, the focus here is on computational descriptively proximities. Briefly, computational descriptive proximity includes algorithms as well as structures such as set intersection, union and closure and proximity space axioms introduced in Peters (2020). Descriptive proximites provide mathematical framework useful in measuring, comparing, and classifying (1) lag structures and threads across frames in the same video or (2) lag structures and threads across frames in different rs-fMRI videos. For example, in terms of (1), ($B_{t}$, inner vortex cycle area) = (100, 100 mm^2^) describes a brain activation subregion in the transversal brain section in frames 10 and 75 in Figure 7. In terms of (2), Let $B_{s},B_{t},B_{c}$ be Betti numbers for the sagittal, transversal and coronal brain regions. The feature vector $\left(B_{s},B_{t},B_{c}\right)$ is used to describe and compare lag threads in frames across different videos. This is an important advantage that accrues from the application of computational descriptive proximities.

### 2.4. Methods

**Figure 2:**
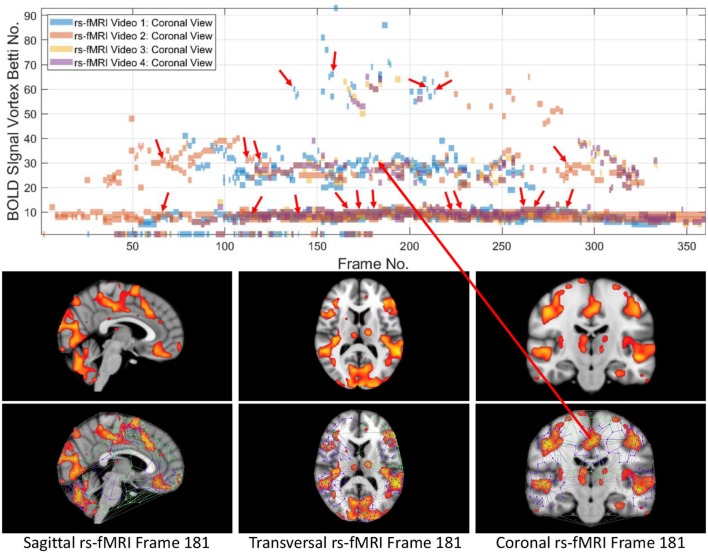
Coronal persistence barcode with Betti numbers as vortex cycle counts in a rs-fMRI video frame (see text for further details).

**Figure 3:**
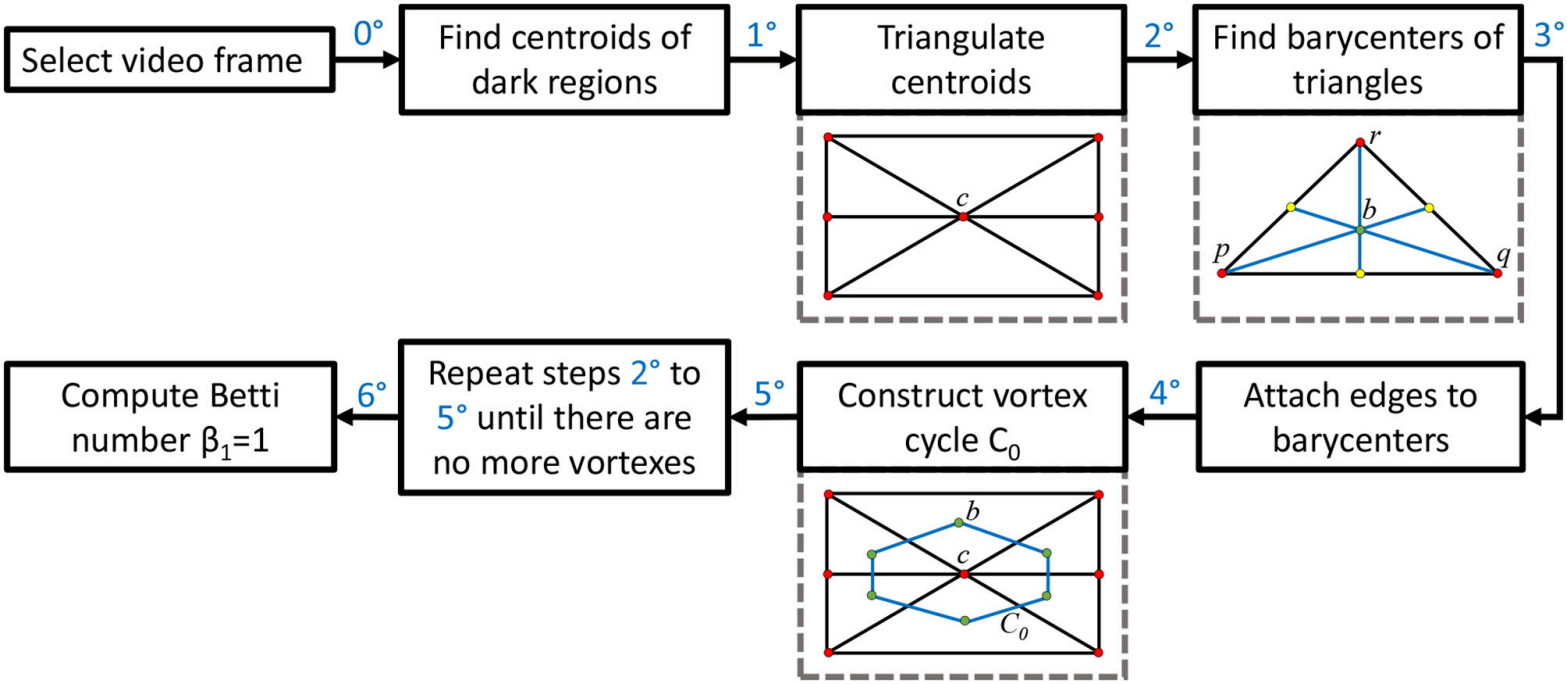
Method flow graph.

This section briefly introduces the method used to derive vortex cycles on triangulated video frames (steps 0 through 5) and their geometric Betti numbers (step 6), which are used to construct persistent barcode for rs-fMRI videos. The fMRI videos (of 688 subjects) used in this work were obtained from the Harvard-MGH Brain Genomics Superstruct Project (Buckner et al., 2014). Each video contains 360 frames that exhibit the propagation of BOLD signals in the sagittal, transversal and coronal sections of the human brain (see, e.g., the middle row of Figure 2). Let *K* be a rs-fMRI video frame. The steps to obtain triangulation vortexes covering the brain activation regions shown in Figure 2 are exhibited in the flow graph in Figure 3.

**0**^**0**^ After selecting a video frame, find the centroids (centers of mass) of the dark background regions. Holes are identified by binarizing each video frame.

**1**^**0**^: Triangulate the centroids of the tiny dark regions inside the brain activation regions in *K*. A sample centroidal triangle is shown in Figure 1.

**2**^**0**^: Find the barycenter of every centroidal triangle △ in *K*. Each barycenter is a voxel representing a high BOLD signal situated between centroids.

**3**^**0**^: Connect the barycenters where there is the greatest concentration of centroidal triangles △s with a common vertex. Recall that the vertex common to a collection of triangles is an example of an Alexandroff nerve (Alexandroff, 1965) (see, e.g., the collection of centroidal triangles with a common vertex covered by the inner vortex cycle in Figure 1). In this work, the focus is on finding maximal Alexandroff nerves.

**4**^**0**^: Construct vortex cycles on the barycenters of centroidal triangles {△} along the boundary of *C*_0_.

**5**^**0**^: Repeat steps **2**^**0**^ through **4**^**0**^ until there are no more vortexes covering subregions containing nonzero BOLD signals. The end result is a collection of connected nesting non-concentric cycles that form a *vortex*. Once these vortexes are generated, the next step is to compute the β_1_ for each vortex that has been found in each of the triangulated video frames. Notice that there is usually more than one vortex in video frame.

**6**^**0**^: Compute Betti number. Count the number of non-single edge (main) cycles in a vortex plus the number of signal edge cycles connected between the main vortex cycles. This process is repeated for each brain section in every frame containing sagittal, trasversal and coronal brain sections in each of the sample rs-fMRI videos.

To construct a Betti number-based persistent barcode, insert a bar in a pictograph (an easy-to-read visualization of brain activity instants accumulated in what is known as a homology barcode Ghrist, 2014), using rs-fMRI video frame number (x-axis) and its corresponding Betti Number (y-axis) (shown in the top half of Figure 1).

## 3. Results

### 3.1. Part 1. Edge Density and Eigen Value Statistics

Since the focus of this study of brain activation in rs-fMRI videos is on the formation of nesting cycles (vortexes) covering the interior brain activation regions, we consider the edge densities and eigenvalues that reflect the levels of connectedness of these activation region cycles.

#### 3.1.1. Edge Density

In this work, edge density quantifies the connectedness in the vortex representation of a brain activation region in a rs-fMRI video frame. Specifically, ***edge density*** is proportional to the product of the number of vortex bridge segments between cycles × the number of cycle vertices. Mathematically, we have

$$\begin{array}{l} \;E=\text{number of bridge segments between vortex cycles}.\\ \;V=\text{number of vortex cycle vertices}.\\ EdgeDensity=2\left[\frac{E}{V\left(V-1\right)}\right]. \end{array}$$

An increase in the number of bridge segments *E* between vortex cycles results in higher edge density. This can happen in the case where there is more than one bridge segment connected to a vortex cycle. An increase in the number of vortex cycle vertices *V* with no change in the number of bridge segments, leads to a lower edge density. This occurs whenever the number of dark regions increase in a brain activation region, which leads to an increase in the number of centroidal triangles. This also leads to an increase in the number of barycenters. Physically, each barycenter pinpoints the location of high intensity in a brain activation region. Each vortex cycle vertex represents a barycenter on brain activation region triangle.

**Figure 4:**
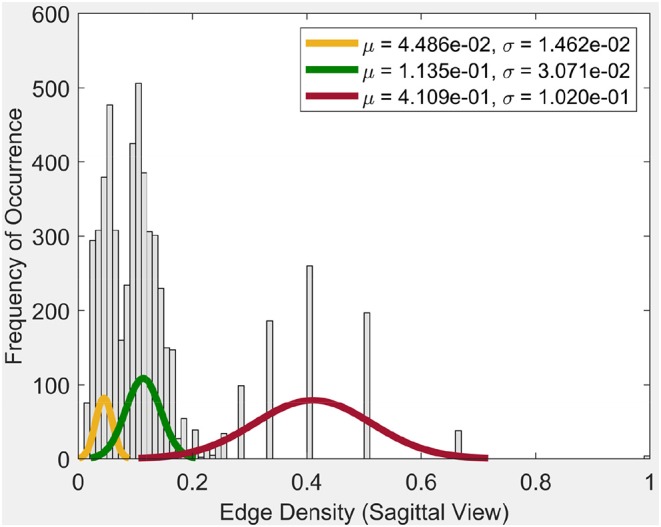
Edge density of sagittal view.

Frequency of occurrence in the plot in Figure 4, for example, is the number of times a certain edge density appears in the cycles. Whenever a certain edge density appears, the plot will show the frequency of occurrence as 1. If it reappears, the frequency of occurrence will be 2. So it will increase the said value for the occurrence by 1, each time it appears.

From the plot for the Sagittal view in Figure 4, it can be seen that each distribution can be represented using three normal distributions. Observe that the left-most normal distribution has a mean μ = 0.04 and a standard deviation σ = 0.014 but it has a lower edge density compared to the other two (middle, right) distributions. The middle normal distribution has μ = 0.11 and a σ = 0.03. This distribution has the highest frequency of occurrence of edges. The right normal distribution has a μ = 0.41 and a σ = 0.1 and has the second highest edge frequency.

When we consider the cycles that are generated by these edge density values, we can observe the following. In order to get a edge density value close to 0.4, there needs to be single cycle with a lower number of vertices. For example, in the case where there is a single cycle with three vertices (i.e., a triangle), we have the edge density of 0.66. This represents the highest value attainable in vortex cycles. Any single or multiple cycles will have a lower edge density than 0.66. In other words, higher the number of cycles and vertices in a cycle; the lower the edge density. Hence, in Figure 4, it can be observed that the highest frequencies of occurrence of edges is for edge densities between 0.1 and 0.2. For further evidence of this, see the plots for transversal and coronal views given in 9 and 10 reported in Appendix A.

#### 3.1.2. Eigenvalue Spectrum

**Figure 5:**
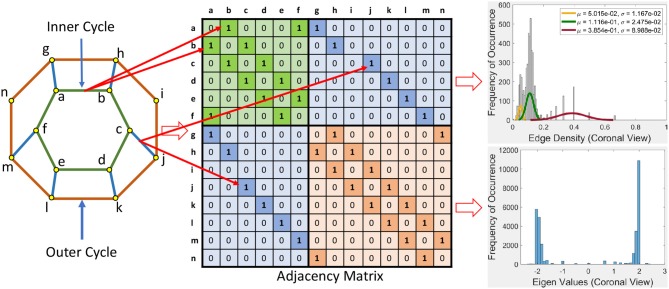
Adjacency matrix representation of cortical vortex Connectedness.

This section briefly introduces an eigenvalue spectrum representation of the eigen values derived from the connectivity relations between cortical vortex cycles. A number λ is an ***eigenvalue*** of a linear transformation *A* (our adjacency matrix), provided there is a vector *x* ≠ 0 so that *A*(*x*) = λ*x*. The vector *x* called an ***eigenvector***. Eigen values are computed using an ***adjacency matrix*** *A*, which is a *n* × *n* square matrix where *n* is the number of vertices in a vortex cycle. Here, an ***eigenvalue spectrum*** is defined by an activation matrix view of the connectedness between vertices in a vortex representation of brain activation regions in rs-fMRI videos. Each adjacency matrix represents the connectivity in vortex cycles. To help visualize the connectivity between vertexes in a vortex, a color-coding scheme is given. That is, the vertices on each vortex cycle edge are color-coded as shown in Figure 5. In this color-coding scheme, sub-matrix (a–f) represents inner cycle vertexes (color-coded Green 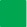). The Sub-matrix (g–n) represents cycle vertexes (color-coded Orange 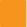). The other two sub matrices represent the bridge segment vertexes (color-coded Blue 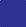).

A bridge segment between vortex cycles with its ending vertices on neighboring cycles has its connectivity represented by a cell containing 1 in the two sub-matrices (color-coded Blue 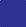) that are not on the main diagonal as illustrated in Figure 5. The bridge segment $\bar{cj}$ is represented by 1s in cells (c,j) and (j,c). If a vortex cycle has an edge between two vertices in locations *i* and *j* the cycle, it is represented symmetrically by 1 in a matrix cell (i,j) and a corresponding cell (j,i). For example if there is a edge between vertices 4 and 6 then cells (4, 6) and (6, 4) is 1 in the corresponding adjacency matrix.

Each eigenvalue plot encapsulates results of the sagittal, transversal and coronal views of brain activations recorded in rs-fMRI videos. Each plot represents 12 separate brain section videos = 3 brain sections × 4 original composite videos (i.e., we have extracted three separate brain section videos from each of the four original composite videos). The separated brain section videos made it possible for us to carry out statistical analysis based on the triangulation of the centroids on holes found in the brain activation regions of each brain section in the reported experimental results recorded in the original four videos.

Example 1. In Figure 5, connectivity between vertices on vortex cycles are color-coded. For instance, the connectivity between vertexes *a* and *b* on the inner cycle in Figure 5 is represented by a green cell 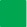 containing a 1 in (*b, a*) (row *b* and column *a*) and by a green cell containing a 1 in (*a, b*) (row *a* and column *b*). 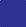


**Figure 6:**
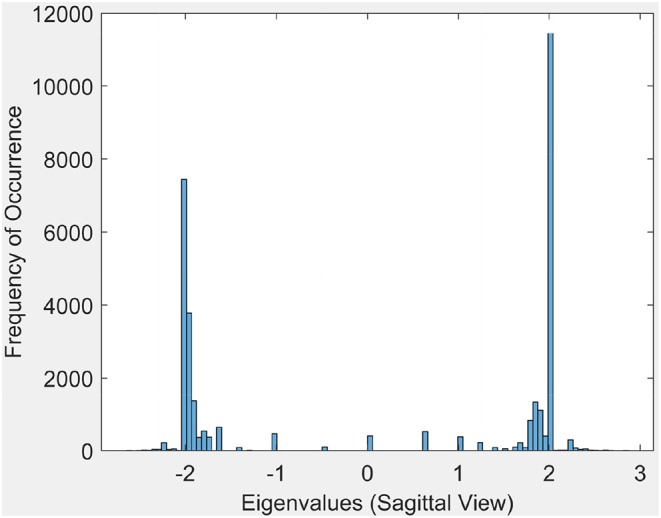
Eigen values for the sagittal view.

Sample results for the eigenvalue spectrum for the sagittal view of brain activation regions is recorded in the plot in Figure 6. Plots of the eigenvalue spectrum for the transversal and coronal views are given in Appendix A.

### 3.2. Part 2. Derivation of Persistent Brain Activation Subregion Signature

Each triangulated BOLD signal propagation subregion has a signature defined by the vector (frame number, cycle count Betti number, inner vortex cycle area). This leads to the production of four triangulated rs-fMRI videos available at Don A. et al. (2019), that are part of the University of Manitoba Vortex Signature Project. Vortexes have been derived from each of the triangulation of the BOLD signal activation regions in each of the brain sections in the video frames in the four rs-fMRI videos from Buckner et al. (2014).

A straightforward outcome of the derived vortexes is a rich source of new means of describing individual BOLD signal activation regions as well as a bridge to various forms of descriptions of lag threads. For example, each vortex has a Betti number (count of the number of connected cycles) and various cycle areas. Each of the three brain regions in each frame in the Harvard Brain Genomics rs-fMRI videos has its own vortex and, consequently, its own Betti number. Typically, each video frame will have more than one Betti number derived from vortexes on the multiple brain activation subregions.

**Figure 7:**
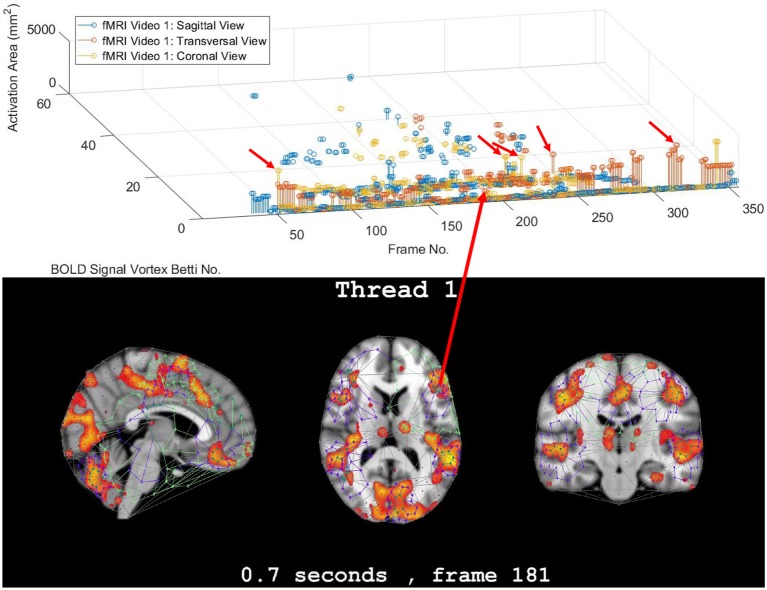
Frame-Betti number-Area for rs-fMRI BOLD signal vortexes on three brain regions.

In this study, the focus is on the area of the inner cycle of a BOLD signal subregion vortex. This is the case, since each inner cycle lies entirely within the interior of an activation subregion. Hence, an inner cycle area is a reliable approximation of a brain activation area. In sum, geometric Betti numbers and inner vortex cycle area help gauge the extent of an activation subregion. Considered either separately or taken together, a vortex on brain activation subregion provides the basis for a subregion signature, i.e., a distinctive characteristic of a brain activation subregion in a rs-fMRI video frame. For example, (frame number, Betti number) = (140, 60), (200, 60), (210, 60) provides a signature for the coronal brain region in frames 140, 200, and 210 in Figure 2. Betti number 60 is an example of a brain activation subregion characteristic that persists over a sequence of video frames. The vector (frame number, Betti number, inner vortex cycle area) = (180, 40, 10 mm^2^) depicts a brain activation subregion in the sagittal brain region in Figure 7. A repetition of the same Betti number for the same brain region across multiple video frames defines a lag thread pattern. For example, $B_{s}=B_{t}=B_{c}=100$ for the sagittal, transversal and coronal brain regions defines a lag thread pattern for multiple frames in Figure 7.

The gaps between the sequences of contiguous bars are important, since gaps in a particular row of pictograph bars indicates rs-fMRI video frames that do not have the same level of brain activity represented by bars in the row. The proximity of the bars (not necessarily contiguous) in a pictograph row call attention to BOLD signals that are close to each other, temporally. Repeated bars in a pictograph row indicate a repeated (persistent) level of brain activity recorded in rs-fMRI video frames. An example of a pictograph row containing multiple, contiguous bars can be seen in row 30 (Betti numbers = 30) in Figure 2. Contiguous bars in a pictograph row indicate the closeness in time of the corresponding brain activity.

Examples of pictograph rows containing multiple, non-contiguous bars can be seen in rows 10 and 60 (Betti numbers = 10 and 60) in Figure 2. A byproduct of the inspection of a sequence of contiguous bars (geometric β_1_ Betti numbers) in a persistent barcode row can lead to the production of a reduced rs-fMRI video containing only video frames with either activation sub-regions with the same Betti number or a new video containing frames with activation sub-regions, each with a different β_1_ (vortex cycle count) Betti number.

### 3.3. Part 3. Confirmation of Highly Reproducible Lag Thread Topography

**Figure 8:**
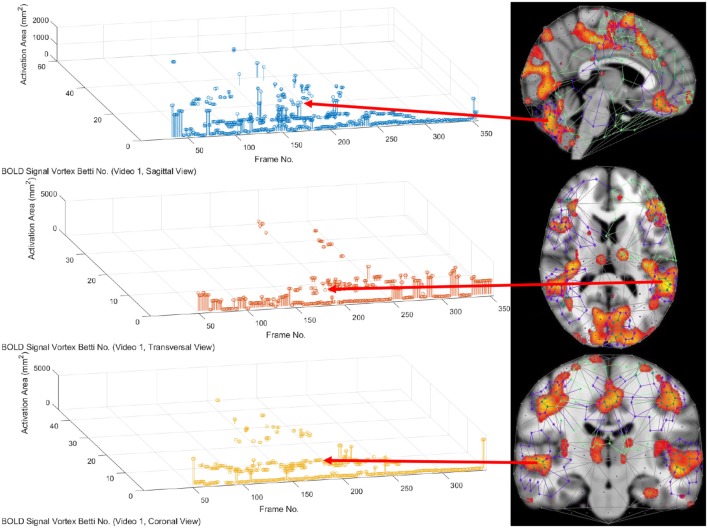
3D persistence barcode for 3 rs-fMRI BOLD signal propagated in brain regions.

From 3D barcodes in Figure 7 as well as in Figure 8, Betti number-area patterns can be detected within frames in the same video. That is, one can find many examples of brain activation subregion Betti number (and corresponding subregion area in a lag thread in one video frame) that are reproduced in a lag thread in a different video frame. In other words, the Betti number-area combination persists across different frames. Many examples of this Betti number-area persistence phenomenon can be detected in the two sample 3D homology barcodes when compared with similar 3D homology barcodes derived from the frames in the videos available at the University of Manitoba Vortex Project at Don A. et al. (2019). These persistent repetitions in the topography detected in different lag threads confirm the observation that there are commonalities in signal propagation within each lag thread (Mitra et al., 2015).

### 3.4. Summary of Findings

This study of the Betti numbers and inner vortex areas of rs-fMRI BOLD signal propagation subregions of the brain confirms and supplements earlier findings given in Mitra et al. (2015). A main result of this study of the persistence of brain activation subregion features confirms the contention that the topography of lag threads is highly reproducible. Starting with the Betti number of connected cycles derived from triangulated brain activation regions found in rs-fMRI videos, it is apparent that the vortexes on brain activation subregions appear over and over in the lag threads across different rs-fMRI video frames. In other words, we find that there are commonalities in BOLD signal propagation within each lag thread.

The question whether intrinsic brain activity contains reproducible temporal sequences is revisited. It is confirmed that a human resting state fMRI (rs-fMRI) contains persistent (repeatable) highly reproducible lag structure. The answer to this question is given twofold. This is done first in terms of Betti numbers that are counts of the number of connected cycles in vortexes on triangulated brain activation subregions. We introduce a 2D persistence pictograph (barcode) that exhibits the appearance, disappearance, and repeated reappearance of Betti numbers across lag threads in sequences of rs-fMRI video frames. In addition, the reproducibility question is also answered in terms of the introduction of a video frame-Betti number-vortex cycle area combination in 3D persistence barcodes that facilitates a check on how often these features of lag threads appear during a rs-fMRI session.

### Concluding Remarks

This study considers Betti numbers that are counts of the number of connected barycentric cycles in vortexes on triangulated brain activation regions. In terms of the area occupied by a vortex on a brain activation subregion, we have only considered the area of the interior of the innermost barycentric cycle of each vortex. Also of interest and of considerable importance is the area in the interior of other cycles that includes the inner vortex cycle. Future work would expand the derivation of persistent barcodes to include the zeroth as well as the oneth Betti numbers. In working toward the approximation of the area of brain activation subregion shapes, the areas in the interior of the other cycles (summing on the innermost vortex cycle area) would be considered.

## Data Availability Statement

The dataset for this study can be found at the following URL: https://drive.google.com/drive/folders/1NxZ1Ydcuhzrdmgav-DxIGrbg-pq5GCI8?usp=sharing.

## Author Contributions

JP designed the materials and methods. AD developed the code and tools. AT and JP analyzed the data and performed the research. SR contributed to the research and writing of the paper.

## Conflict of Interest

The authors declare that the research was conducted in the absence of any commercial or financial relationships that could be construed as a potential conflict of interest.
