# Supplementary material for: Topological View of Flows Inside the BOLD Spontaneous Activity of the Human Brain
Source: Front Comput Neurosci. 2020 Apr 22;14:34. doi: 10.3389/fncom.2020.00034 (PMC7189216; doi:10.3389/fncom.2020.00034)
Supplement: Supplementary file 1 [file Data_Sheet_1.pdf]

Topological View of Flows inside the BOLD Spontaneous Activity of the Human Brain

Arjuna P.H. Don [1], **James F. Peters** [1,*], **Sheela Ramanna** [2] **and Arturo Tozzi** [1,3]

[1] *Computational Intelligence Laboratory, University of Manitoba, WPG, MB, R3T 5V6, Canada*
[2] *Applied Computer Science, University of Winnipeg, WPG, MB, R3B 2E9, Canada*
[3] *University of North Texas, Department of Physics, Denton, TX 76201, USA*

Correspondence*:
James F. Peters
james.peters3@umanitoba.ca

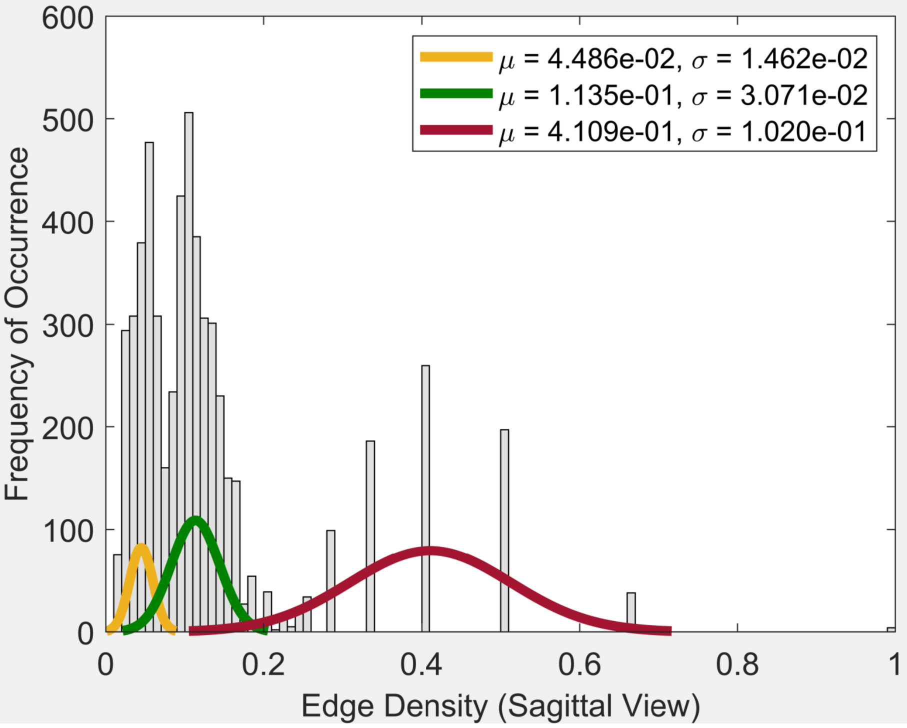

Figure 1. Edge Density of Sagittal View

1 APPENDIX A. FURTHER STATISTICAL ANALYSIS RESULTS

This appendix gives further statistical analysis results for a topology of data approach in the study of cortical lag threads recorded in rs-fMRI videos and reported in this paper.

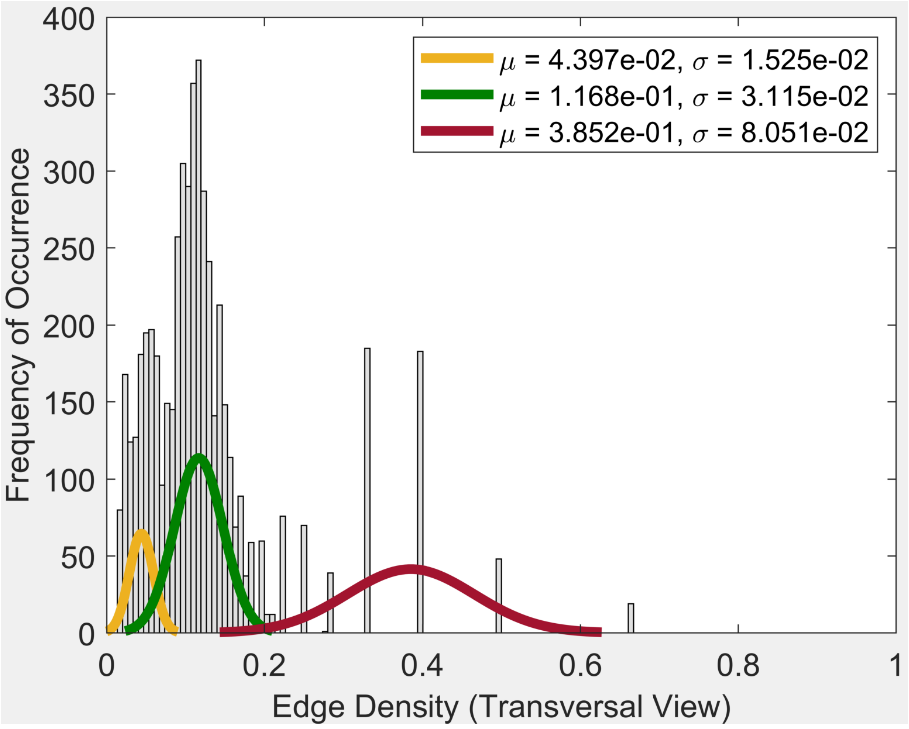

Figure 2. Edge Density of Transversal View

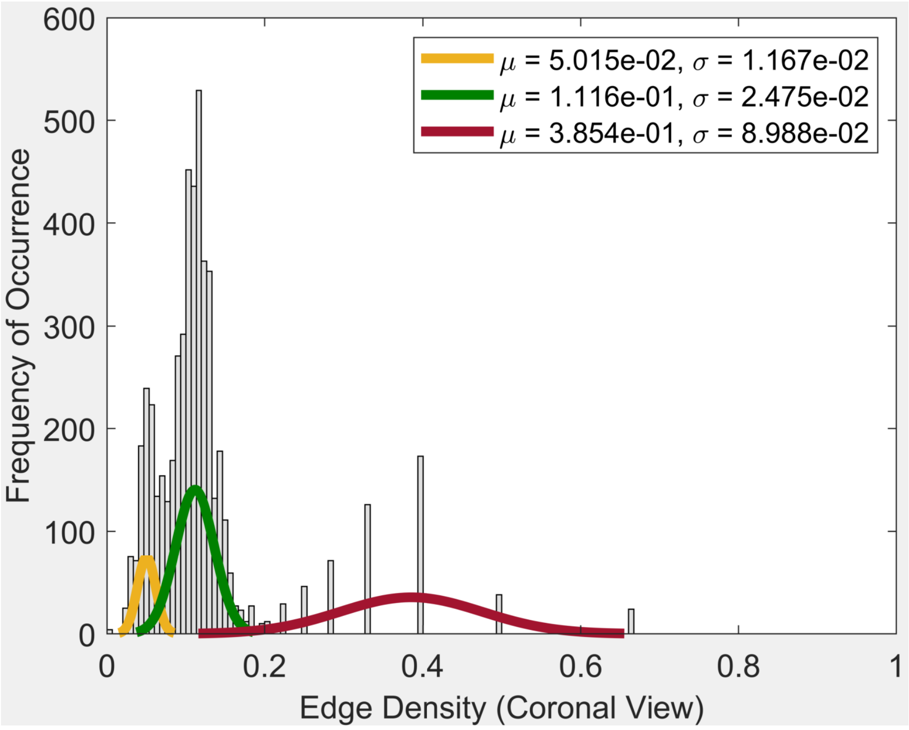

Figure 3. Edge Density of Coronal View

1.1 Edge Density Revisited

Figures 2 and 3 show the distribution of the edge density of the transversal and coronal brain sections for all 4 threads.

Frequency of occurrence in the plot in Fig. 2, for example, is the number of times a certain edge density appear in the vortex cycles on the triangulated transversal activation regions. Each time a particular first appearance of an edge density appears in the vortex cycles, the plot will show the frequency of occurrence as 1. The frequency of occurrence of an edge density is incremented by 1 each time that edge density appears in the vortexes on the rs-fMRI video frames.

From Figures 1, 2 and 3, it can observed that the highest frequencies of occurrence of edges is for edge densities between 0.1 and 0.2.

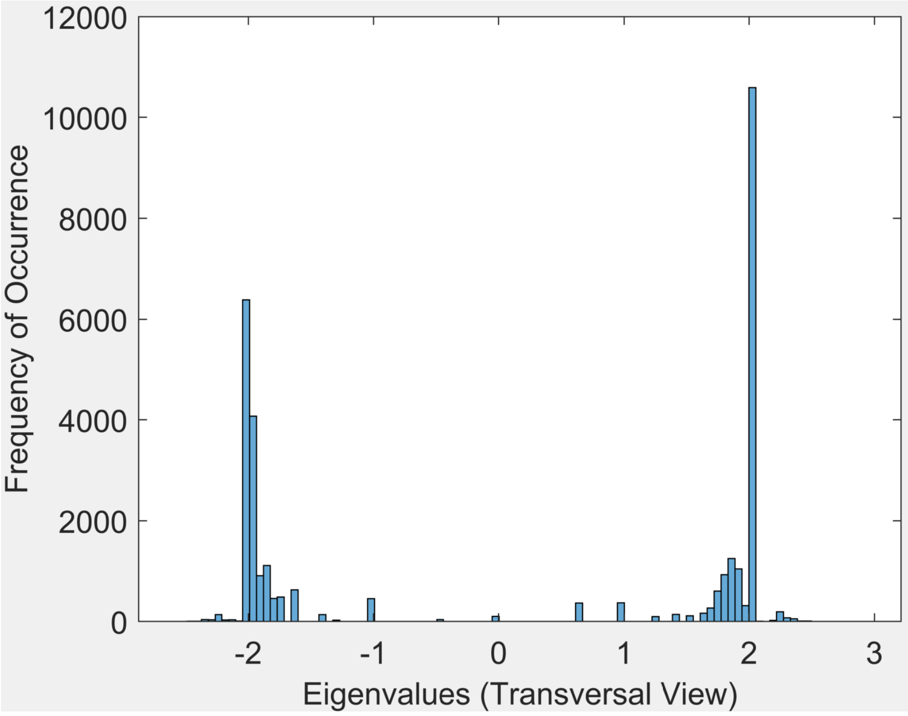

Figure 4. Eigen Values for the Transversal View

1.2 Eigenvalue Spectrum Revisited

Further eigenvalue results are given in the plots in Fig. 4 and 5 for the transversal and coronal views of brain activity.

Each of these eigenvalue plot encapsulates results for the transversal and coronal views of brain activations recorded in rs-fMRI videos. Notice that each plot represents 12 separate brain section videos = 3 brain sections $\times$ 4 original composite videos.

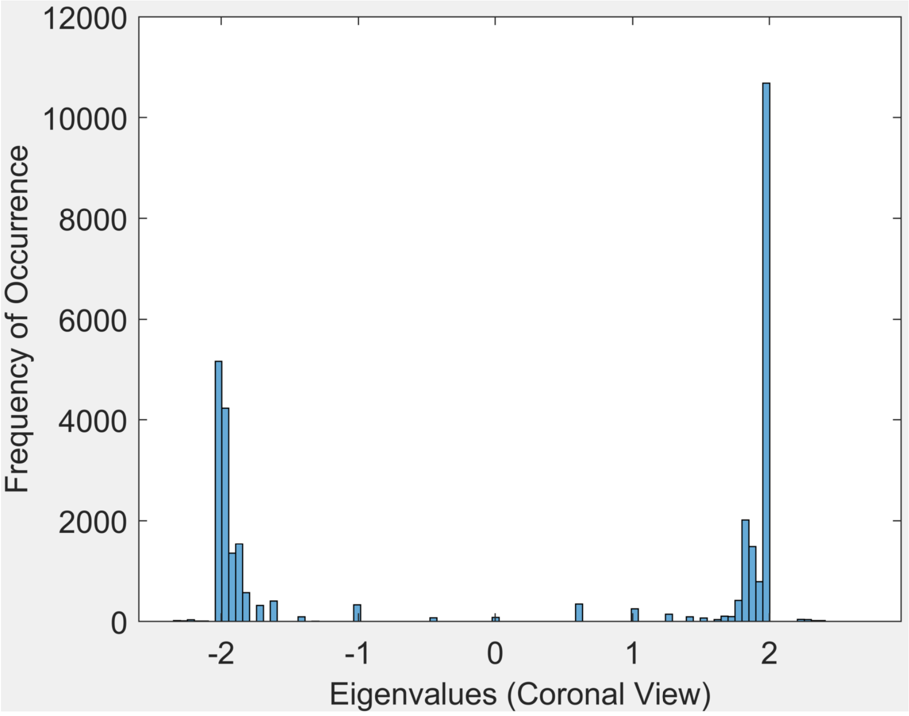

Figure 5. Eigen Values for the Coronal View
