# Supplementary material for: Topological View of Flows Inside the BOLD Spontaneous Activity of the Human Brain
Source: Front Comput Neurosci. 2020 Apr 22;14:34. doi: 10.3389/fncom.2020.00034 (PMC7189216; doi:10.3389/fncom.2020.00034)
Supplement: Supplementary file 2 [file Data_Sheet_2.pdf]

frontiers

# Topological View of Flows inside the BOLD Spontaneous Activity of the Human Brain

**Arjuna P.H. Don [1], James F. Peters [1,*], Sheela Ramanna [2] and Arturo Tozzi [1,3]**

[1] *Computational Intelligence Laboratory, University of Manitoba, WPG, MB, R3T 5V6, Canada*
[2] *Applied Computer Science, University of Winnipeg, WPG, MB, R3B 2E9, Canada*
[3] *University of North Texas, Department of Physics, Denton, TX 76201, USA*

Correspondence*:
James F. Peters
james.peters3@umanitoba.ca

## 1 APPENDIX B. BETTI NUMBERS

The results for two types of Betti numbers are reported here, namely, algebraic and geometric.

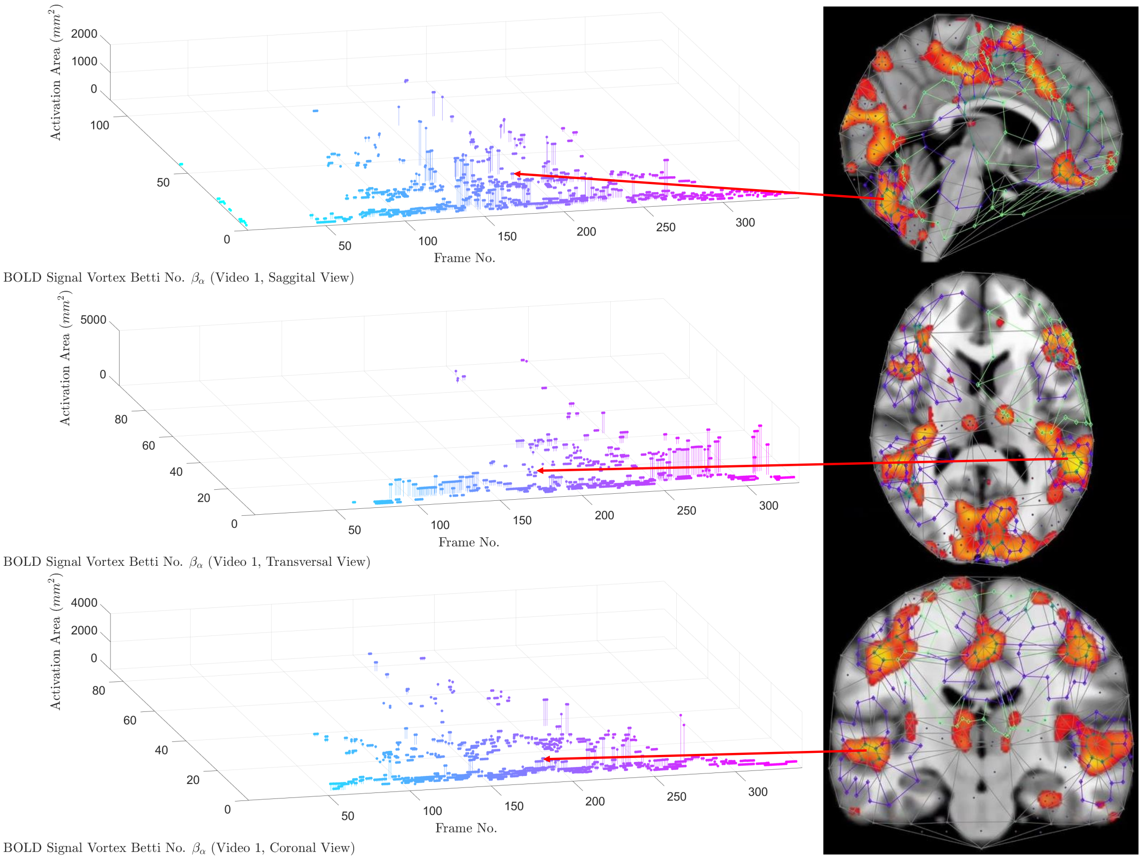


**Figure 1.** Three views of $\beta_\alpha$ persistence

### 1.1 Algebraic Betti Numbers

An ***algebraic Betti number*** (denoted by $\boldsymbol{\beta_\alpha}$) is a count of the number of generators in the free Abelian group (Munkres, 1984, §1.4) representation of a vortex covering a brain activation region.

A ***vortex*** is a collection of nesting, usually non-concentric, path-connected, barycentric, intersecting cycles (Peters, 2020, §2.2). In this work, a vortex provides a mechanism for quantifying the number of pathways found within brain activation regions. A ***cycle*** $E$ contains vertexes so that each pair of vertexes $p, q$ in the cycle $E$ is path-connected, *i.e.*, there is a sequences of edges leading from vertex $p$ to vertex $q$ in the cycle. The vertexes in barycentric cycles are the barycenters (intersection of the median lines) of triangles.

Thanks to the presence of edges (called bridge segments or filament cusps (Peters, 2020, §4.11)) attached between vertexes on neighbouring cycles, we can always find a path between any vertex on vortex cycle and any other vertex either on the same cycle or on a different cycle. Each vertex on a connecting edge between vortex cycles plays the role of a generator in a free Abelian group representation of a brain activation region vortex. A connecting edge vertex can be thought of as either the entrance or the exit of a bridge between cycles. A Betti number $\beta_\alpha$ quantifies this view of generators in terms of counts of the number of vertexes on the bridge segments between brain activation vortex cycles. For this reason, the label **bridge vertex** is used to identify one of the vertexes on a bridge segment in Fig. 2.

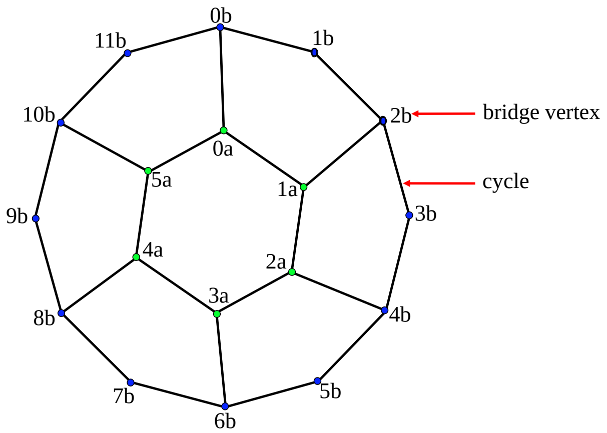


**Figure 2.** Sample brain activation area vortex

EXAMPLE 1. *In Fig. 2, there are 6 bridge segments (denoted by br) between a pair of cycles (outer cycle with vertexes labelled* $0b, 1b, \ldots$ *and inner cycle with vertexes labelled* $0a, 1a, \ldots$*. In this case,* $\beta_\alpha = 2 \times br = 2 \times 6 = 12$.

Next, we briefly explain the group theory underlying algebraic Betti numbers. Recall that a group is a nonempty set $V$ (for vortex) equipped with a binary operation (represented here with a + (read traverse or move over a sequence of edges between vortex cycle vertices)), so that each vertex $p$ of $V$ has an inverse $-p$ with $p + (-p) = 0$ (*i.e.*, no traversal or movement occurs) and $p + q = q + p$ (Abelian property). That is, traversing the edges from $p$ to $q$ in the vortex can always be followed by a traversal of the edges from $q$ to $p$, which takes us back to where we started. A zero move is the identity element of the group. For example, $p + 0$ reads 'no traversal occurs at $p$'. A group ***generator*** is a vertex $\boldsymbol{a}$ that represents $k$ vertices $a_1, \ldots, a_k$ on the ends of the edges in a vortex cycle. Since all traversals of paths between cycles depends on the endpoints of vortex bridge segments, the generators of a vortex group are identified with bridge

segment vertices.

Example 3 Generators $\mathbf{0}\boldsymbol{a}$ and $\mathbf{0}\boldsymbol{b}$ (bridge segment endpoints) represent the inner and outer cycles on the vortex in Fig. 2. A move from vertex $2b$ on the outer cycle to vertex $3a$ on the inner cycle is defined by a sequence of moves between these vertices, i.e.,

$$3a = 2b + 1b + 0b + 0a + 1a + 2a + 3a,$$

which is a sequence of moves over the path between $2b$ and $3a$. ■

In a free Abelian group representation of a vortex, each vertex in the nerve can be written as a summation of the generating elements. The number of generators in such a group is the **rank** of the group. In our case, the rank of a group representation of a vortex with $N$ bridge segments between cycles equals $2 \times N$.

Theorem (Peters, 2020, §4.13, p. 212) A vortex that is a cell complex has a free Abelian group representation.

Example 4
A sample vortex is shown in Fig. 2. The vertexes on the edges attached between the inner and outer cycles are the generators of the free Abelian group representations of these vortexes. ■

Example 5
A sample application of algebraic Betti numbers in tracking the persistence of $\beta_\alpha$ on triangulated brain activation areas is shown in Fig. 1. This application exhibits the changes of inner vortex area (activation area) relative to the $\beta_\alpha$ Betti Number values for each rs-fMRI video frame. When compared to the $\beta_1$ Betti numbers, these have a higher spread. This indicates that bridge vertexes (generating elements) are more sensitive to changes in brain activation areas than $\beta_1$ Betti number vortex cycle counts computed for triangulated activation areas in each rs-fMRI video frame. ■

## 1.2 Geometric Betti Numbers

***Geometric Betti numbers*** (denoted by $\beta_0, \beta_1, \beta_2$) are counts of the occurrence of different cell stuctures Zomorodian (2001) that appear in the triangulation of activated brain regions. The Betti number $\beta_0$ is a count of the total number of occurrences of several elementary cell structures that are the basic building blocks in cell complexes, namely, 0-cells (vertexes), 1-cells (edges) and 2-cells (filled triangles) covering brain activation areas. A **cell complex** is a collection elementary cells attached to each other by edges or by having one or more common vertices. We have limited this study of triangulated brain activation areas to the persistence of $\beta_1$ counts over sequences of rs-fMRI video frames, since $\beta_0$ counts of elementary cell complexes and and $\beta_2$ counts of holes (dark regions in brain activation regions) are already part of the structure of path-connected vortex cycles counted by the $\beta_1$ Betti number.

Example 6
The persistence of Betti number $\beta_1$ over video frame sequences of an rs-fMRI video is shown in the 3D stem plots in Fig.4. These 3D stem plots exhibit changes in inner vortex areas covering brain activation regions

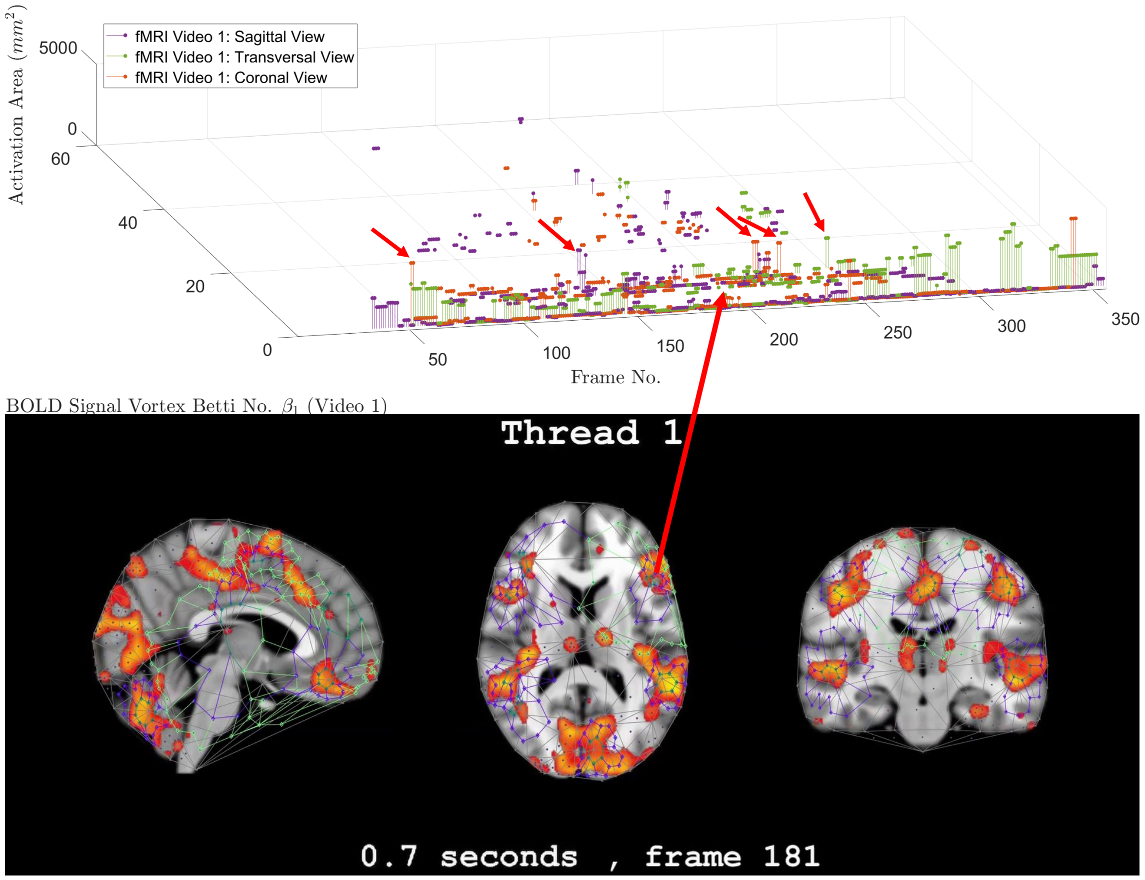


**Figure 3.** Frame-Betti number $\beta_1$-Area for an rs-fMRI BOLD signal vortexes on 3 brain regions

for each frame relative to changing values of the $\beta_1$ Betti number. A sample vortex is shown in Fig. 2. ■

Example 7

An expanded view of the persistence of Betti number $\beta_1$ cycle counts over video frame sequences for the sagittal, transversal and coronal rs-fMRI videos are shown in the 3D stem plots in Fig.4. These 3D stem plots give a detailed view of changes in inner vortex areas covering brain activation regions for each frame relative to changing values of the $\beta_1$ Betti number. ■

## REFERENCES

Munkres, J. (1984). *Elements of algebraic topology* (Menlo Park, CA: Addison-Wesley Publishing Company). ix+454pp, ISBN: ISBN: 0-201-04586-9

Peters, J. (2020). *Computational geometry, topology and physics of digital images with applications. Shape complexes, optical vortex nerves and proximities* (Cham, Switzerland: Springer Nature). xxv+440pp.,ISBN 978-3-030-22191-1/hbk,`https://doi.org/10.1007/978-3-080-22192-8`

Zomorodian, A. F. (2001). *Computing and Comprehending Topology: Persistence and Hierarchical Morse Complexes*. Ph.D. thesis, University of Illinois at Urbana-Champaign,Chicago,USA, Department of Computer Science

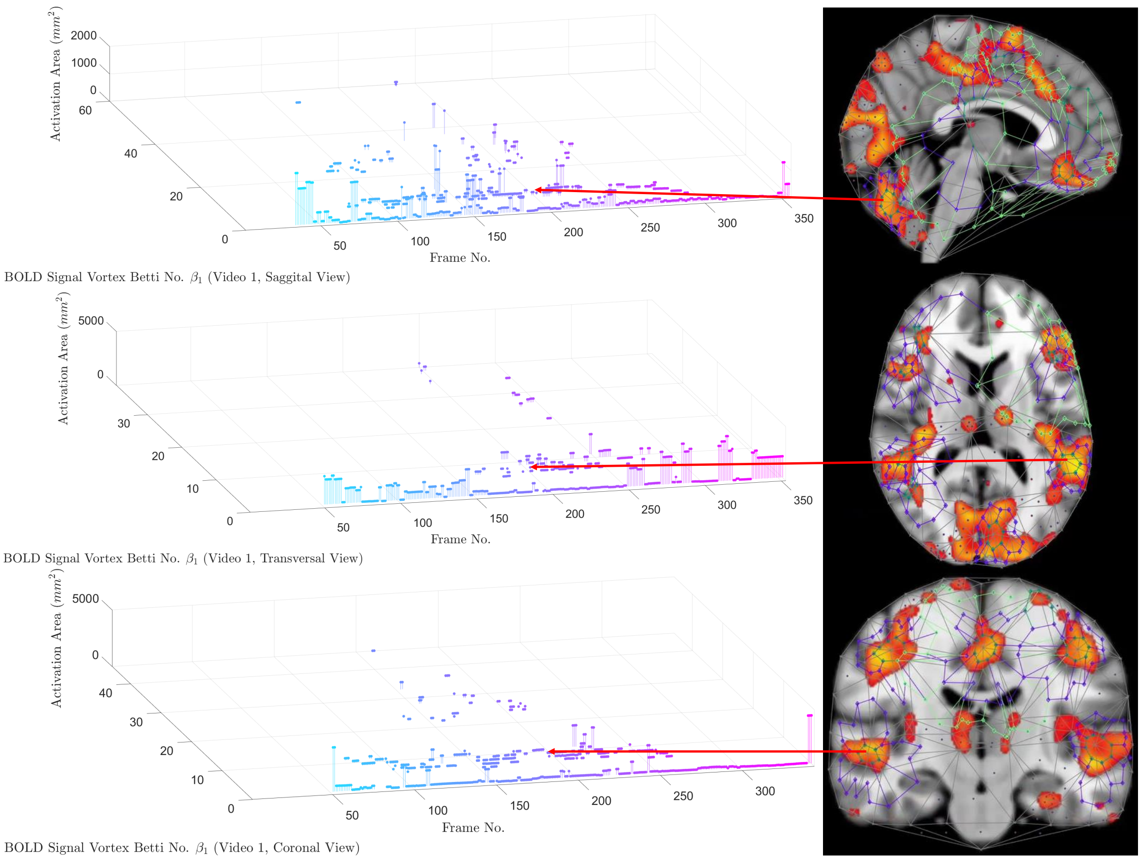


**Figure 4.** 3D stem plots for rs-fMRI BOLD activation regions
